# Predicting osteoradionecrosis risk in patients with locoregionally advanced nasopharyngeal carcinoma undergoing concurrent chemoradiotherapy: The value of the CARWL index

**DOI:** 10.17305/bb.2024.11155

**Published:** 2024-09-22

**Authors:** Nulifer Kilic Durankus, Efsun Somay, Sibel Bascil, Sukran Senyurek, Duriye Ozturk, Ugur Selek, Erkan Topkan

**Affiliations:** 1 Department of Radiation Oncology, School of Medicine, Koc University, Istanbul, Türkiye; 2 Department of Oral and Maxillofacial Surgery, Faculty of Dentistry, Baskent University, Ankara, Türkiye; 3 Department of Periodontology, Faculty of Dentistry, Baskent University, Ankara, Türkiye; 4 Department of Radiation Oncology, Faculty of Medicine, Afyonkarahisar Health Sciences University, Afyonkarahisar, Türkiye; 5 Department of Radiation Oncology, Faculty of Medicine, Baskent University, Adana, Türkiye

**Keywords:** C-reactive protein-to-albumin ratio, weight loss, CARWL index, osteoradionecrosis, nasopharyngeal carcinoma

## Abstract

Osteoradionecrosis (ORN) is a severe complication that can arise in patients with nasopharyngeal carcinoma due to the aggressive nature of chemoradiotherapy treatment. The purpose of our study was to assess the utility of the recently introduced CARWL index, which integrates the C-reactive protein-to-albumin ratio (CAR) and significant weight loss (SWL), in predicting the risk of ORN in patients with locoregionally advanced nasopharyngeal cancer (LA-NPC) undergoing concurrent chemoradiotherapy (CCRT). We conducted a retrospective cohort analysis on 304 patients with LA-NPC treated with CCRT. Patients were categorized into CARWL index groups based on CAR (cut-off: 3.0) and SWL (weight loss > 5% over the past six months): CARWL-0 (CAR < 3.0, SWL ≤ 5%), CARWL-1 (CAR < 3.0 with SWL > 5% or CAR ≥ 3.0 with SWL ≤ 5%), and CARWL-2 (CAR ≥ 3.0 and SWL > 5%). The primary endpoint was the incidence of ORN in each CARWL index group. At a median follow-up of 67.2 months, 28 patients (9.2%) developed ORN. The incidence of ORN was 2.1%, 9.4%, and 16.3% in the CARWL-0, CARWL-1, and CARWL-2 groups, respectively (*P* < 0.001). Multivariate analysis identified smoking status (HR: 2.58, *P* ═ 0.034), N-stage (HR: 1.96, *P* ═ 0.008), T-stage (HR: 1.84, *P* ═ 0.017), pre-CCRT tooth extraction status (HR: 5.81, *P* < 0.001), post-CCRT tooth extraction status (HR: 6.82, *P* < 0.001), mandibular V55.8 Gy (HR: 6.12, *P* < 0.001), and CARWL score (HR: 5.67, *P ═* 0.002) as significant predictors of ORN. The CARWL index is a reliable predictive tool for evaluating the risk of ORN in LA-NPC patients undergoing CCRT. If further validated, its use in clinical settings could aid in the early identification of high-risk patients and enable the implementation of personalized preventive strategies.

## Introduction

Nasopharyngeal cancer (NPC) is relatively rare globally, but it exhibits significant geographic variation in incidence. The highest rates are observed in Southeast Asia and North Africa, with annual cases reaching 20–30 per 100,000 individuals. In contrast, the incidence of NPC in Western countries is typically less than one case per 100,000 annually [1]. The main factors contributing to NPC include genetic predisposition, Epstein–Barr virus (EBV) infection, dietary habits (such as consuming salted fish), and environmental exposures [2].

The standard treatment for locoregionally advanced NPC (LA-NPC) consists of radiotherapy (RT), chemotherapy, or a combination of both, known as concurrent chemo-RT (CCRT). Recent advances in RT techniques, particularly intensity-modulated RT (IMRT), have significantly improved treatment outcomes and reduced severe side effects. However, treatment-related toxicities, especially chronic effects, remain a major concern for many patients [3]. These chronic side effects can manifest months or even years after completing treatment. One notable side effect is osteoradionecrosis (ORN), a condition where the jawbone deteriorates due to radiation damage. ORN is a serious complication that can severely impact the patient’s quality of life [4].

The primary risk factor for developing ORN is radiation dose–volume parameters related to the mandible. Research consistently shows that higher radiation doses to the mandible increase the risk of ORN [5]. Despite advances in RT and preventive strategies, ORN still affects 5%–15% of patients undergoing RT or CCRT [6, 7]. Therefore, adhering to pre-established dose–volume constraints for the mandible is crucial to reducing ORN risks [8].

ORN develops through radiation-induced damage to endothelial cells, leading to reduced blood flow and subsequent bone necrosis. The condition is characterized by hypocellularity, hypoxia, hyperfibrosis, and high levels of inflammation. Inflammation plays a key role in ORN progression by triggering the release of pro-inflammatory cytokines, such as IL-1β, tumor necrosis factor-alpha (TNF-α), and NF-κB, which aggravate tissue damage and hinder healing [9]. Recent research has highlighted the potential of inflammatory biomarkers in predicting ORN risk.

Notably, these biomarkers include the neutrophil–lymphocyte ratio (NLR), platelet–lymphocyte ratio (PLR), monocyte–lymphocyte ratio (MLR), and the pan-immune-inflammation value (PIV) [10–14].

In addition to blood-based biomarkers, other factors, such as biochemical markers and patient characteristics, have also been linked to ORN risk. For instance, elevated C-reactive protein (CRP) levels and decreased albumin (Alb) levels, as measured by the CRP-to-Alb ratio (CAR), have been associated with an increased risk of ORN and poor overall prognosis [15, 16]. This underscores the significance of systemic inflammation, compromised immunity, and poor nutrition in relation to patient outcomes. Weight loss, driven by inflammation and tumor metabolism, further complicates the prognosis. Significant weight loss (SWL), defined as a loss of more than 5% body weight over six months, has been associated with elevated inflammatory markers and lower Alb levels [17]. This correlation increases the risk of treatment-related complications.

Recently, Topkan et al. [18] developed a composite predictive tool called the CARWL index, which combines CAR and SWL. This index was used to predict outcomes in patients with stage IIIC non-small cell lung cancer (NSCLC) undergoing CCRT. The study found that higher CARWL scores were associated with poorer survival. This was the first study to show that patients with higher CARWL scores had significantly worse overall survival and progression-free survival, emphasizing the potential of the CARWL index in clinical settings [18]. However, despite its potential, the CARWL index has not been studied in relation to CCRT-related toxicity. Therefore, this retrospective study aims to assess whether the CARWL index can predict ORN in LA-NPC patients treated with CCRT.

## Patients and methods

### Study population

We analyzed clinical data from patients with LA-NPC who underwent oral and dental examinations prior to CCRT between January 2007 and December 2022. Inclusion criteria required histopathological confirmation of nasopharyngeal squamous cell carcinoma, age ≥ 18 years, an Eastern Cooperative Oncology Group (ECOG) performance status of 0–1, a body mass index (BMI) ≥ 18.5 kg/m^2^, and clinical or radiological confirmation of LA-NPC based on the American Joint Committee on Cancer (AJCC) 8th edition (T1-2N1-3M0 or T3-4N0-3M0). In addition, records of RT, chemotherapy, dental examinations, and pre-CCRT levels of CRP, Alb, and weight loss in the preceding six months were required for inclusion.

Patients were excluded if they had mandibular involvement from the primary tumor or lymph nodes, a previous diagnosis of ORN, had taken steroids within 30 days of CCRT, or had systemic inflammatory conditions (e.g., rheumatologic diseases, nephritis, viral hepatitis, respiratory disorders, or other chronic inflammatory diseases). These exclusion criteria aimed to minimize bias related to pre-existing immune and inflammatory conditions.

### Treatment protocol

All patients initially received three-dimensional conformal radiation therapy (3D-CRT) until 2010, after which simultaneous integrated boost IMRT (SIB-IMRT) was adopted. Target volumes were defined using computed tomography (CT), 18-fluorodeoxyglucose positron emission tomography/CT (18F-FDG-PET-CT), and/or magnetic resonance imaging (MRI) of the primary tumor and neck [19]. Treatment plans followed established protocols, with high-risk planning target volumes (PTVs) receiving 70 Gy, intermediate-risk PTVs receiving 59.4 Gy, and low-risk PTVs receiving 54 Gy, delivered in 33–35 daily fractions, five days a week.

For patients undergoing 3D-CRT, treatment was administered in phases with separate plans for each phase of radiation. Concurrent cisplatin chemotherapy was administered every 21 days at a dose of 75–80 mg/m^2^. Two additional cycles of cisplatin-based chemotherapy were given as adjuvant therapy post-RT. Supportive treatments, including analgesics, antiemetics, and nutritional support, were provided based on individual patient needs.

### Baseline oral and dental examinations

In accordance with the American Dental Association (ADA) and the U.S. Food and Drug Administration (FDA) guidelines, all patients underwent comprehensive oral and dental examinations prior to the start of CCRT [20]. These evaluations included oral hygiene instructions and treatment for periodontal, restorative, and endodontic issues for salvageable teeth. Teeth deemed unsalvageable were extracted based on the following criteria: severe root resorption, pulp, periodontal, or periapical disease, root caries involving more than half of the root, impacted teeth with follicular cysts, or residual roots. All clinical and radiographic examinations were performed by experienced oral and maxillofacial radiologists and surgeons.

Panoramic radiographs were taken for all patients using a digital system (J Morita, Veraviewepocs 2D, Kyoto, Japan) with settings at 70 kVp, 10 mA, and an exposure time of 9 s. Patients were positioned according to the manufacturer’s instructions.

### Creation of CARWL score groups

The CARWL index was calculated by combining the CAR and SWL data for each patient. CAR was determined as CRP/Alb, based on blood tests taken on the first day of CCRT. The percentage of weight loss over the six months preceding CCRT was calculated using patients’ weight on the first day of CCRT compared to their weight six months prior. Following Fearon et al.’s Delphi criteria, SWL was defined as a weight loss >5% during this period [21].

### Follow-up assessments

All patients followed a standardized oral health care protocol, including professional oral hygiene instruction and follow-up dental care. After completing CCRT, patients received professional oral care every three months for two years, followed by six-monthly assessments. These follow-up visits included periodontal, restorative, and endodontic procedures as needed. Radiographic examinations were performed based on clinical requirements, adhering to established guidelines for ORN detection [22].

ORN was diagnosed based on clinical and radiological criteria: irradiated bone that fails to heal over three months, without evidence of persistent, recurrent, or metastatic disease [23, 24].

### Ethical statement

This study adhered to the ethical standards outlined by Baskent University Medical Faculty’s Institutional Review Board (IRB NO: DKA19/39-B). All patients provided written informed consent before undergoing CCRT, allowing the use of their clinical data, blood samples, and test results for research purposes. The study complied with the ethical principles of the Declaration of Helsinki and Good Clinical Practice guidelines, including any revisions.

### Statistical analysis

The primary objective was to assess the correlation between pre-CCRT CARWL scores and the incidence of ORN post-CCRT. Continuous variables were expressed as medians and ranges, while categorical variables were reported as percentages. Statistical comparisons between groups were performed using chi-square tests, Student’s *t*-tests, or Spearman correlation analysis, as appropriate.

**Table 1:** Baseline patient demographics and treatment characteristics

**Characteristic**	**All patients**	**CARWL-0**	**CARWL-1**	**CARWL-2**	***P* value**
	**(*N* ═ 304)**	**(*N* ═ 96)**	**(*N* ═ 116)**	**(*N* ═ 92)**	
Median age, years (range)	55 (18–78)	55 (18–77)	56 (20–76)	54 (22–78)	0.83
*Age group, years (%)*					
< 65 years	237 (77.9%)	74 (77.1%)	92 (79.3%)	71 (77.2%)	0.91
≥ 65 years	67 (22.1%)	22 (22.9%)	24 (20.7%)	21 (22.8%)	
*Gender, n (%)*					
Male	206 (67.8%)	63 (65.6%)	80 (69.0%)	63 (68.5%)	0.86
Female	98 (32.2%)	33 (34.4%)	36 (31.0%)	29 (31.5%)	
*Smoking status, n (%)*					
Yes	199 (65.5%)	64 (66.7%)	77 (66.4%)	58 (63.0%)	0.84
No	105 (34.5%)	32 (33.3%)	39 (33.6%)	34 (37.0%)	
*Alcohol consumption, n (%)*					
Yes	129 (42.4%)	45 (46.9%)	46 (39.7%)	38 (41.3%)	0.38
No	175 (57.6%)	51 (53.1%)	70 (60.3%)	54 (58.7%)	
*ECOG performance status, n (%)*					
0	169 (55.6%)	52 (54.2%)	62 (53.4%)	55 (59.8%)	0.57
1	135 (44.4%)	44 (45.8%)	54 (46.6%)	37 (40.2%)	
*T-stage, n (%)*					
T1-2	81 (26.6%)	26 (27.1%)	30 (25.9%)	25 (27.2%)	0.71
T3-4	223 (73.4%)	70 (72.9%)	86 (74.1%)	67 (72.8%)	
*N-stage, n (%)*					
N0-1	65 (21.4%)	21 (21.9%)	24 (20.7%)	20 (21.7%)	0.54
N2-3	239 (78.6%)	75 (78.1%)	92 (79.3%)	72 (78.3%)	
*Pre-CCRT tooth extraction, n (%)*					
Absent	8 (2.6%)	2 (2.1%)	2 (1.7%)	4 (4.3%)	0.49
Present	296 (97.4%)	94 (97.9%)	114 (98.3%)	88 (95.7%)	
Median tooth extraction time to CCRT, days (range)	15 (10–24)	15 (10–24)	14 (11–23)	15 (10–22)	0.81
*Post-CCRT tooth extraction, n (%)*					
Absent	70 (23.0%)	22 (22.9%)	27 (23.3%)	21 (22.8%)	0.62
Present	234 (77.0%)	74 (77.1%)	89 (76.7%)	71 (77.2%)	
*Concurrent chemotherapy cycles, n (%)*					
1	59 (19.4%)	19 (19.8%)	22 (19.0%)	18 (19.6%)	035
2-3	245 (80.6%)	77 (80.2%)	94 (81.0%)	74 (80.4%)	
*Adjuvant chemotherapy cycles, n (%)*					
0	80 (26.3%)	16 (16.7%)	50 (43.1%)	14 (15.2%)	0.67
1-2	224 (73.7%)	80 (83.3%)	66 (56.9%)	78 (84.8%)	
MMD, Gy (range)	36.8 (10.1–59.3)	35.4 (10.8–58.2)	31.9 (12.7–56.8)	36.2 (10.9–57.5)	0.55
*MMD group, n (%)*					
< 36.8 Gy	163 (53.6%)	51 (53.1%)	62 (53.4%)	50 (54.3%)	0.48
≥ 36.8 Gy	141 (46.4%)	45 (46.9%)	54 (46.6%)	42 (45.7%)	
*Mandibular V55.8 Gy group, n (%)*					
< 35.2%	143 (47.0%)	45 (46.9%)	55 (47.4%)	43 (46.7%)	0.51
≥ 35.2%	161 (53.0%)	51 (53.1%)	61 (52.6%)	49 (53.3%)	

CARWL: C-reactive protein-to-albumin ratio and significant weight loss; CCRT: Concurrent chemoradiotherapy; ECOG: Eastern Cooperative Oncology Group; MMD: Mean mandibular dose; V36.8 Gy: Volume receiving 36.8 Gray or higher.

Receiver operating characteristic (ROC) curve analysis was used to determine the optimal cut-off value for CAR, where the J-index was maximized. This threshold was used to categorize patients into high-risk and low-risk ORN groups. Kaplan–Meier curves were employed to estimate the time to ORN onset, while a multivariate Cox proportional hazards model was used to evaluate the relationship between patient, disease, treatment variables, and ORN risk. All tests were two-sided, with *P* values < 0.05 considered statistically significant. To minimize the risk of false-positive findings in subgroup analyses, Bonferroni corrections were applied to *P* values when comparing three or more groups, with a threshold of <0.017 for significance in such cases.

## Results

In this retrospective study, we evaluated 304 patients diagnosed with LA-NPC who underwent radical CCRT. The median age of the cohort was 55 years (range: 18–78 years), with a male predominance of 67.8%. Most patients (55.6%) had an ECOG performance status score of 0. Histologically, 85% of patients were classified as World Health Organization (WHO) Type II/III. Tumor stages were T1-2 in 26.6% and T3-4 in 73.4% of cases, while nodal stages were N0-1 in 21.4% and N2-3 in 78.6% (Table 1).

**Figure 1.**
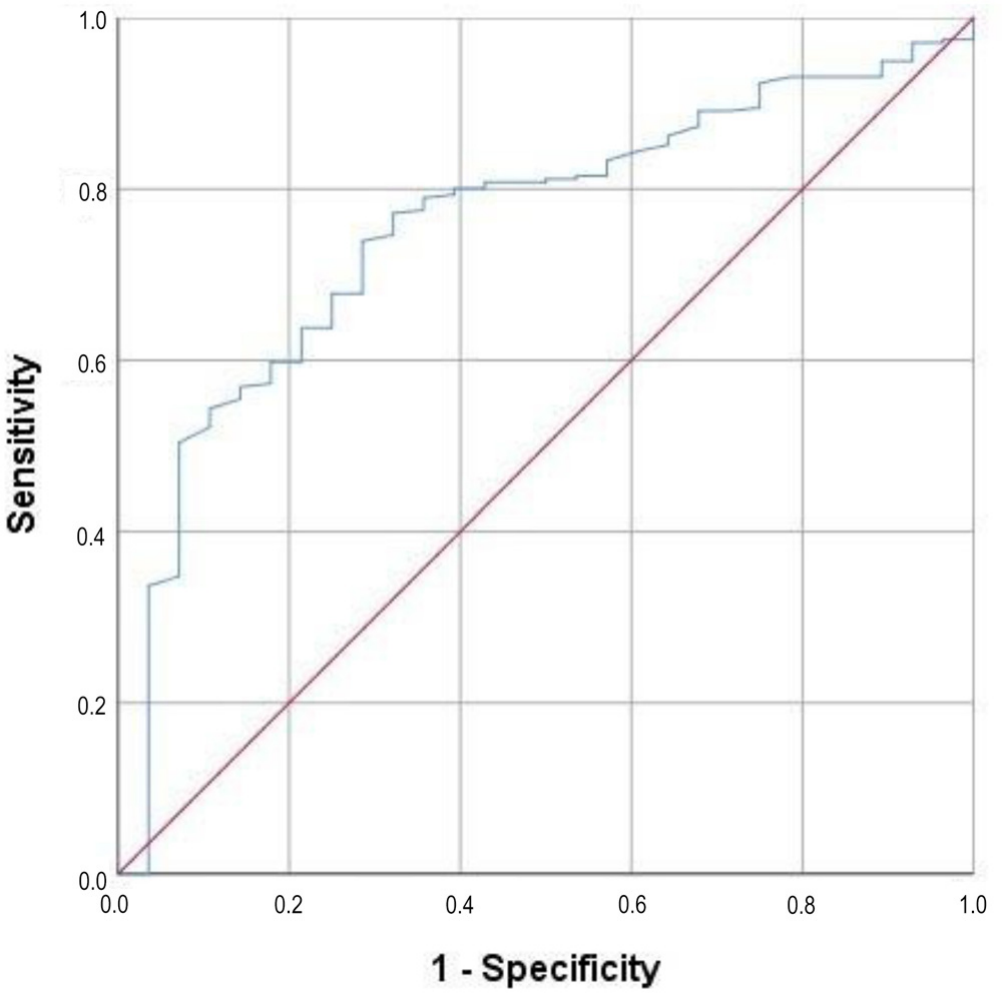
**The outcomes of a receiver operating characteristic curve analysis examining the correlation between CAR and significant WL index (CARWL) and osteoradionecrosis rates (CARWL cutoff: 3.04; area under the curve: 75.7%; sensitivity: 73.9%; specificity: 71.8%, J-index: 0.457).** CAR: C-reactive protein-to-albumin ratio; WL: Weight loss.

The CAR index was determined using ROC curve analysis, with a significant cut-off point of 3.04 that divided the patients into two ORN risk groups: CAR < 3.040 and CAR ≥ 3.0 (area under the curve [AUC]: 75.7%; sensitivity: 73.9%; specificity: 71.8%; and J-index: 0.457) (Figure 1). For simplicity in further analyses, we rounded the cut-off to 3.0. Thus, patients were grouped into CAR < 3.0 (*N* ═ 153) and CAR ≥ 3.0 (*N* ═ 151). SWL was defined as a body weight loss of >5% within six months before CCRT, in accordance with previous studies by Topkan et al. [18] and Evans et al. [25]. Based on this, the study cohort was split into two SWL groups: SWL ≤ 5% (*N* ═ 162) and SWL > 5% (*N* ═ 142).

By following the original stratification methods [18], four possible CARWL index groups were created: CARWL-0: CAR < 3.0 and SWL absent; CARWL-1: CAR < 3.0 and SWL present, CARWL-2: CAR ≥ 3.0 and SWL absent, and CARWL-3: CAR ≥ 3.0 and SWL present. However, because the ORN rates between the CARWL-1 and CARWL-2 groups were not significantly different (8.6% vs 9.8%; *P* ═ 0.67), we merged these groups into a single category. The final CARWL groups were thus: **CARWL-0** (*N* ═ 96), **CARWL-1** (*N* ═ 116), and **CARWL-2** (*N* ═ 92) (Table 2).

At a median follow-up of 67.2 months (range: 10.2–116.2 months), 28 patients (9.2%) developed ORN, with the median time from CCRT to ORN being 21 months (range: 15–34 months). Despite similar distributions of patient, disease, and treatment factors, the incidence of ORN significantly increased across the CARWL groups, rising from 2.1% in the CARWL-0 group to 9.4% in the CARWL-1 group and 16.3% in the CARWL-2 group (*P* < 0.001) (Table 3).

Univariate and multivariate analyses were conducted to identify significant predictors of ORN. In the univariate analysis, the following factors were associated with a significantly higher risk of developing ORN: ever-smoking (vs never smoking; *P* ═ 0.021), N2-3 nodal stage (vs N0-1; *P* ═ 0.003), pre-CCRT tooth extractions (vs no extractions; *P* < 0.001), post-CCRT tooth extractions (vs no extractions; *P* < 0.001), mandibular V55.8 Gy > 35.2% (vs V55.8 Gy < 35.2%; *P* < 0.001), and higher CARWL score (2 vs 1 vs 0; *P* < 0.001).

**Figure 2.**
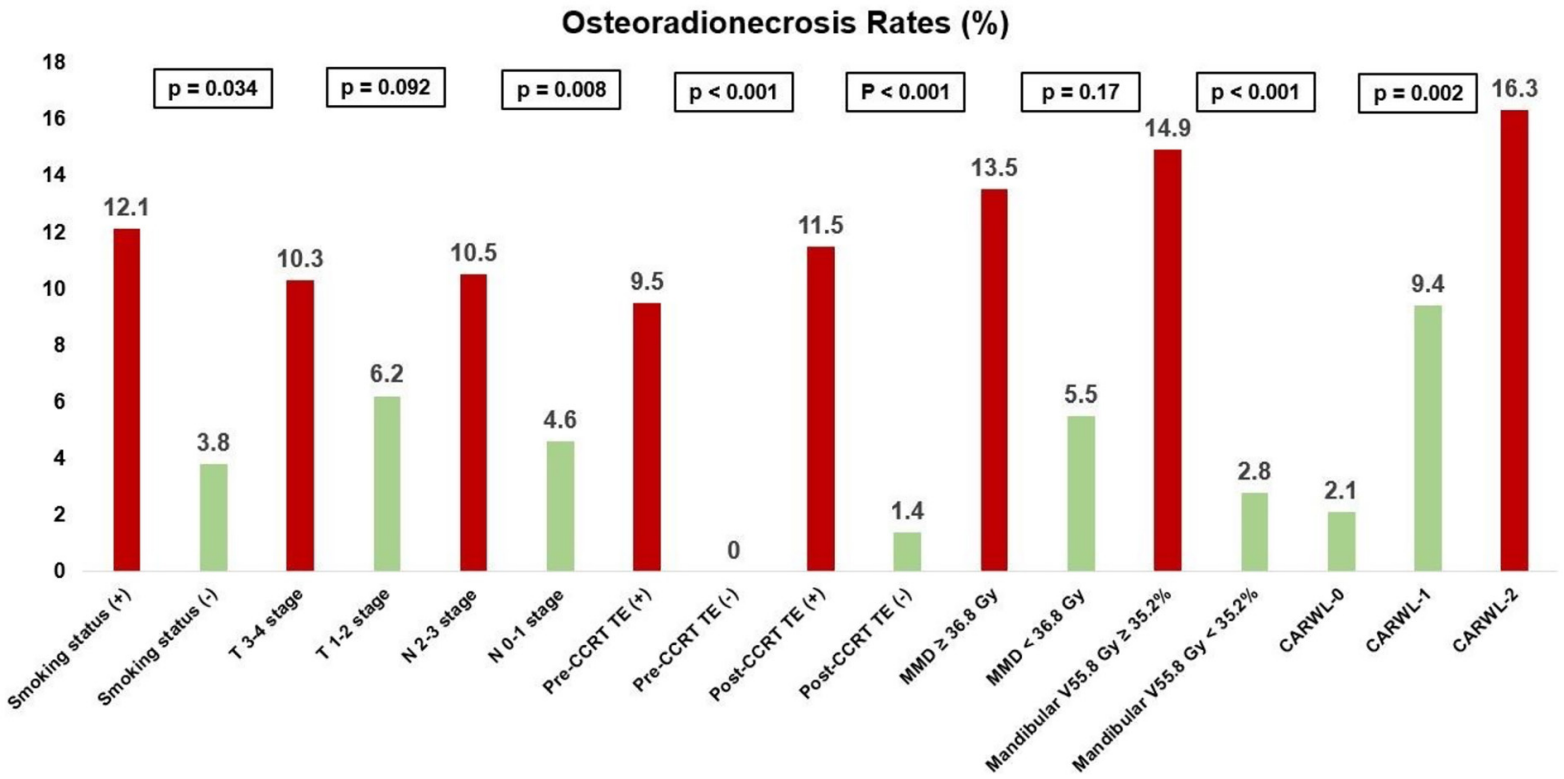
**Bar graph displaying the frequency of ORN across different variables that demonstrated significance in the multivariate analysis.** The significant predictors of ORN, with their corresponding *P* values, are as follows: smoking status (yes vs no), N-stage (N2-3 vs N0-1), pre-CCRT tooth extraction (present vs absent), post-chemoradiotherapy tooth extraction (present vs absent), mean mandibular dose ( ≥ 36.8 Gy vs < 36.8 Gy), mandibular V55.8 Gy group (≥ 35.2% vs < 35.2%), CARWL score (CARWL-2 vs CARWL-1 vs CARWL-0). T-stage: Tumor stage; N-stage: Nodal stage; CCRT: Concurrent chemoradiotherapy; TE: Tooth extraction; MMD: Mean mandibular dose; CARWL: C-reactive protein-to-albumin ratio and significant weight loss; ORN: Osteoradionecrosis.

These variables were further analyzed in a multivariate Cox proportional hazards model, which confirmed their independent predictive value for ORN (*P* < 0.05 for all factors) (Table 3 and Figure 2).

**Table 2:** Definition of CARWL score groups

**Group**	**Definition**
CARWL-0	CAR < 3.0 and SWL absent
CARWL-1	CAR < 3.0 and SWL present or CAR ≥ 3.0 and SWL absent
CARWL-2	CAR > 3.0 and SWL present

CARWL: Combination of CAR and SWL; CAR: C-reactive protein-to-albumin ratio; SWL: Weight loss > 5% in the previous six months.

**Table 3:** Results of univariate and multivariate analyses

**Characteristic**	**All patients**	**ORN (%)**	**Univariate**	**Multivariate**	**HR**
	**(*N* ═ 304)**	**(*N* ═ 28)**	***P* value**	***P* value**	**(95% CI)**
*Age group, N (%)*					
< 65 years	237	23 (9.7)	0.58	–	–
≥ 65 years	67	5 (7.5)			
*Gender, n (%)*					
Male	206	20 (9.7)	0.49	–	–
Female	98	8 (8.2)			
*Smoking status, n (%)*					
Yes	199	24 (12.1)	0.021	0.034	2.58 (1.64–4.71)
No	105	4 (3.8)			
*Alcohol consumption, n (%)*					
Yes	129	15 (11.6)	0.23	–	–
No	175	13 (7.4)			
*ECOG performance status, n (%)*					
0	169	16 (9.5)	0.76	–	–
1	135	12 (8.9)			
*T-stage, n (%)*					
T1-2	81	5 (6.2)	0.041	0.092	–
T3-4	223	23 (10.3)			
*N-stage, n (%)*					
N0-1	65	3 (4.6)	0.003	0.008	1.96 (1.23–3.07)
N2-3	239	25 (10.5)			
*Pre-CCRT tooth extraction, n (%)*					
Absent	8	0 (0.0)	<0.001	<0.001	5.81 (3.78–9.17)
Present	296	28 (9.5)			
*Post-CCRT tooth extraction, n (%)*					
Absent	70	1 (1.4)	<0.001	<0.001	6.82 (3.12–10.78)
Present	234	27 (11.5)			
*Concurrent chemotherapy cycles, n (%)*					
1	59	5 (8.5)	0.62	-	-
2-3	245	23 (9.4)			
*Adjuvant chemotherapy cycles, n (%)*					
0	80	7 (8.8)	0.79	–	–
1-2	224	21 (9.4)			
*MMD group, n (%)*					
< 36.8 Gy	163	9 (5.5)	0.009	0.17	1.84 (1.32–2.51)
≥ 36.8 Gy	141	19 (13.5)			
*Mandibular V55.8 Gy group, n (%)*					
< 35.2%	143	4 (2.8)	<0.001	<0.001	6.12 (4.17–14.81)
≥ 35.2%	161	24 (14.9)			
*CARWL score group*					
0	96	2 (2.1)	0.001	0.002	5.67 (3.02–8.89)
1	116	11 (9.4)			
2	92	15 (16.3)			

CARWL: C-reactive protein-to-albumin ratio and significant weight loss; CCRT: Concurrent chemoradiotherapy; ECOG: Eastern Cooperative Oncology Group; MMD: Mean mandibular dose; V55.8 Gy: Volume receiving 55.8 gray or higher.

## Discussion

This study demonstrates that the CARWL index, which integrates the CRP to Alb ratio and SWL, is a potent predictive tool for assessing the risk of ORN in patients with LA-NPC undergoing CCRT. Our findings show that higher CARWL scores are strongly associated with increased ORN incidence, with patients in the highest CARWL group exhibiting an ORN incidence of 16.3%, compared to only 2.1% in the lowest group. These results suggest that the CARWL index could be invaluable in clinical practice for identifying high-risk patients and implementing targeted preventive strategies to improve patient outcomes.

The most notable finding of our study was the first demonstration of a robust connection between pre-CCRT CARWL scores and ORN incidence rates. Specifically, we observed a progressive increase in ORN incidence following CCRT corresponding to CARWL scores: 2.1%, 9.4%, and 16.3% in the CARWL-0, CARWL-1, and CARWL-2 score categories, respectively (*P* < 0.001). Figure 2 depicts a noticeable escalation in ORN rates from CARWL-0 to CARWL-1 and CARWL-2 score groups. Namely, the higher the CARWL score, the higher the ORN risk. This finding substantiates the effectiveness of the CARWL index in identifying patients at elevated risk for ORN through multivariate analysis. From a clinical perspective, this risk stratification could be instrumental in avoiding less advanced RT planning and delivery techniques, ultimately leading to reduced mandibular doses and ORN rates. Moreover, this stratification may prompt more stringent follow-up protocols in the CARWL-1 and CARWL-2 score groups, enabling timely diagnosis of ORN and the initiation of necessary treatment measures, which may prevent or reduce the need for debilitating surgical interventions in these patient groups.

While it is challenging to precisely explain the direct correlation between higher CARWL scores and increased ORN risks without comparable studies, it is still possible to propose logical explanations by considering the components of the CARWL index and their impact on ORN development and progression. The CARWL index consists of two main factors: CAR and SWL. CRP is an acute-phase reactant protein that increases in response to inflammation. Even a single inflammatory stimulus is enough to trigger a rapid increase in CRP synthesis in the liver, leading to elevated CRP levels. However, this CRP-induced release of TNF-α and interleukin-6 (IL-6) in response to inflammation decreases serum Alb levels due to increased breakdown and reduced production in hepatocytes [26]. Hence, CRP and Alb levels are inversely connected. Decreased Alb levels also indicate malnutrition and a weakened immune system, which may significantly contribute to ORN development and progression. Furthermore, heightened inflammation and elevated CAR levels may damage blood vessels, resulting in insufficient nourishment and mandibular hypoxia—a key characteristic of ORN. Although further research is required in this area, current data supports a plausible correlation between a higher CAR value (i.e., a combination of CRP and Alb) and increased ORN risk [16].

Regarding SWL, body weight loss of more than 5% over six months may raise ORN risk by indicating ongoing health deterioration and poor nutritional status, likely on the path to cancer cachexia, where bodily needs are not met for proper functioning or wound healing. This renders patients more susceptible to complications like ORN [17]. Consequently, although further corroborative research is needed, a higher CARWL score may indicate a weakened immune and nutritional status and exacerbated systemic inflammation, as our current research suggests. By integrating these inflammatory and nutritional markers, the CARWL index provides a comprehensive assessment of a patient’s risk profile for ORN, underscoring its potential utility in guiding clinical decisions and tailoring treatment plans.

In addition to the CARWL index, this study identified five other factors independently associated with significantly increased ORN rates following CCRT. These include smoking, N2-3 stage, pre- and post-CCRT tooth extractions, and mandibular V55.8 Gy > 35.2%. Smoking is a well-established risk factor for ORN due to its exacerbation of radiation-induced vascular damage, leading to heightened tissue necrosis [9]. Similarly, advanced N-stage correlates with higher ORN risk, likely due to the unavoidable exposure of larger mandibular volumes to higher radiation doses, particularly in level I-IIA neck nodes due to their proximity to the mandible [3]. The association between pre- and post-CCRT dental extractions and ORN aligns with existing literature, showing that trauma to irradiated bone from dental procedures markedly escalates the likelihood of ORN development [24]. Regarding dose–volume relationships, the observed connection between mandibular V55.8 Gy > 35.2% and increased ORN risk supports existing studies suggesting a higher likelihood of ORN in patients whose larger mandibular volumes receive higher doses. These findings underscore the complex convergence of various factors in ORN development and highlight the need for a comprehensive approach to risk evaluation and mitigation.

The CARWL index examined in this study possesses several characteristics of an ideal biological marker for accurately distinguishing between low-risk and high-risk patients for a specific endpoint [27]. Specifically, an ideal marker should be cost-effective, easily quantifiable, reproducible across diverse populations and laboratories, and safe for integration into clinical practice [28]. The CARWL index meets these criteria, as it only requires a simple biochemistry test for CAR calculation and determination of weight change over six months before CCRT—without incurring additional costs. Furthermore, the CARWL index provides comprehensive information regarding a patient’s immune, inflammatory, and nutritional status in a single measurement. This characteristic makes it less prone to biases than markers encompassing only one or two parameters. Therefore, if validated, our results suggest that the CARWL index could serve as a reliable biological marker for predicting ORN rates and guiding preventive measures in LA-NPC patients undergoing CCRT, and possibly in other head and neck cancers.

The present study is strengthened by the use of standardized staging, a uniform CCRT protocol, pre- and post-treatment oral health assessments, and consistent diagnostic criteria for ORN. However, certain limitations should be considered. First, the study’s single-center retrospective design introduces the potential for selection bias, which is common in studies of this nature. Additionally, our analysis was based on a single set of CARWL data before CCRT, which may compromise the precision of our findings, as CARWL component levels could vary significantly during and after CCRT due to changes in tumor burden, inflammation, immunity, and nutrition. For instance, a different CAR threshold measured during or after CCRT, combined with further weight loss exceeding 5%, might demonstrate stronger correlations with ORN incidence. Finally, by not investigating potential correlations between CARWL score groups and other biomarkers (e.g., proinflammatory or fibrosis-inducing cytokines and chemokines), we may have missed a more precise understanding of the relationship between higher CARWL scores and elevated ORN rates. Therefore, our findings should be considered preliminary until validated by further comprehensive research that addresses these concerns directly.

## Conclusion

In conclusion, this study suggests that the CARWL index is a novel and reliable tool for predicting ORN risk in LA-NPC patients undergoing CCRT. With further validation, the CARWL index could become a key component of personalized cancer care, enabling more precise risk stratification and tailored treatment plans that improve patient outcomes.

## Data Availability

The data supporting this study’s findings is available from the corresponding author, Efsun Somay, upon a reasonable special request.
